# A Review of Peripheral Vascular Intervention Technologies

**DOI:** 10.3390/jcm15166199

**Published:** 2026-08-11

**Authors:** Yau Ching Yun, Laura S. Kraemer, John R. Hallsten, James Friend, Ann Gaffey

**Affiliations:** 1 Medically Advanced Devices Laboratory, Department of Mechanical and Aerospace Engineering, Jacobs School of Engineering and Department of Surgery, School of Medicine, University of California San Diego, 9500 Gilman Drive, La Jolla, CA 92093, USAjamesfriend@wustl.edu (J.F.); 2 Center for Medical Device Engineering and Biomechanics, Department of Surgery, School of Medicine, University of California San Diego, 9500 Gilman Drive, La Jolla, CA 92093, USA; 3 Department of General Surgery, Naval Medical Center San Diego, 38400 Bob Wilson Drive, San Diego, CA 92134, USA; 4 Department of Vascular Surgery, School of Medicine, University of California San Diego, 9500 Gilman Drive, La Jolla, CA 92093, USA; 5 Department of Mechanical Engineering and Materials Science, McKelvey School of Engineering, Washington University in St. Louis, 1 Brookings Lane, St. Louis, MO 63130, USA

**Keywords:** peripheral artery disease, critical limb threatening ischemia, catheter, fabrication, endovascular treatment

## Abstract

**Background/Objectives**: Chronic limb-threatening ischemia (CLTI) is the end stage of peripheral arterial disease (PAD) and leads to severe morbidity and mortality. With an increase in diabetes mellitus (DM) and an aging population, tibial artery CLTI is becoming more prevalent. Conventional endovascular devices are often limited by both length and size, highlighting the need for improved endovascular microcatheters. **Methods**: Here we provide a narrative review of CLTI in tibial disease and current treatment options, highlighting current endovascular techniques, the mechanics and challenges of current catheters, and call attention to the need for novel micro-steerable catheters. **Results**: Although PAD endovascular treatments have advanced over the past several decades, active tip steering for tibial and intrahospital microcatheters remains limited and without prospective clinical outcome data despite broader advances in peripheral endovascular therapy and catheter tip steering for larger vessels. Challenges in developing steerable microcatheters include the geometry and mechanics of the catheter’s tip on a microscale (50 µm–900 µm). Micro-fabrication methods have shown early promise in creating intricate microstructures; however, challenges remain in precise fabrication and assembly. Further research and development of novel microcatheters for the treatment of tibial disease is needed, as larger steerable catheters have been shown to improve patient outcomes with decreased procedure time, fluoroscopy time, required contrast, and procedural complications. **Conclusions**: The refinement of steerable micro catheters in tibial vessel intervention would expand current endovascular treatment and diagnostic capabilities, likely improving patient outcomes.

## 1. Introduction on PAD with Progression to End-Stage PAD or CLTI

**Figure 1:**
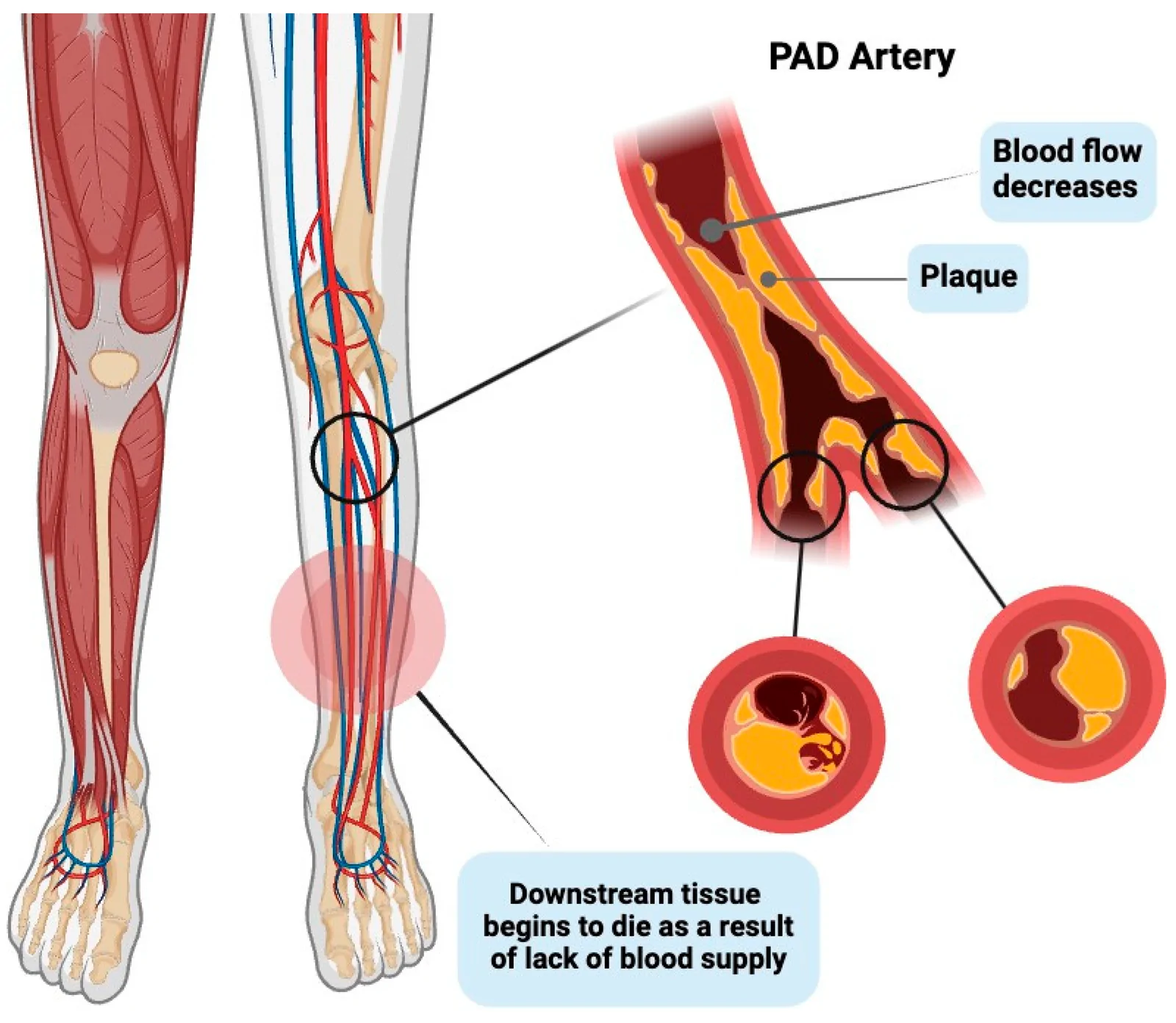
Peripheral Arterial Disease (PAD) is a circulatory problem in which the blood vessels are narrowed, causing reduced or blocked blood flow to the extremities.

Peripheral arterial disease (PAD) refers to conditions affecting blood vessels outside the heart—typically in the legs or lower extremities—and is linked to an elevated risk of cardiovascular disease, cerebrovascular events such as stroke, myocardial infarction (MI), and even death [1]. PAD is well known in the vascular surgery community, although historically it has been underappreciated compared to other atherosclerotic diseases, such as coronary artery disease and cerebrovascular disease [2]. More recently, PAD has gained recognitions as an important cardiovascular disease with over 230 million people worldwide being affected. In 1995, PAD affected 8–12 million people in the United States. Multiple factors contribute to the development of PAD, including smoking, obesity, diabetes, and metabolic syndrome. With an increasing prevalence of many of these comorbidities, it is not surprising that the prevalence of PAD is steadily increasing and is expected to reach 15–24 million in the United States by 2030 [3]. Despite increased awareness of PAD, a significant need remains for improved management and treatment options [2,4,5,6]. Much of this need revolves around chronic limb-threatening ischemia (CLTI), the most advanced form of the disease. It is characterized by severe progression of PAD, where lack of arterial blood flow to the lower extremities results in rest pain, wounds, tissue loss, gangrene, and increased risks of amputation or death (Figure 1) [2,7,8].

Approximately 100,000 people are diagnosed each year with CLTI [9]. They are at increased risk for concomitant cardiovascular disease, such as coronary artery disease or carotid artery disease [10]. Major lower extremity amputations occur at an alarming rate of 15–20% at one-year post-diagnosis in patients with CLTI [11]. One-year mortality rates vary anywhere from 15 to 40% and often increase beyond 50% four to five years after the initial diagnosis, especially in patients with severe CLTI [11]. The economic burden of CLTI is significant. The estimated annual cost per patient is $11,553 [12] and the total estimated annual cost to Medicare is around $12 billion dollars [5,11]. The combination of its large clinical burden and poor prognosis makes CLTI a major threat to public health [5]. Decreasing the rate of “life or limb” decisions is essential for maximizing the quality of life for those living with PAD, as many experience numerous challenges post-amputation, including reduced independence, difficulty returning to the workforce, and mental health challenges [13].

The incidence of CLTI has been reported to be around 11% of patients with PAD; however, some argue this is an underestimation as many administrative codes (e.g., ICD-9-CM) may miss or exclude complex CLTI presentations due to coding variability [14,15,16]. This has been reported to produce up to a 25% underestimation of true prevalence [14,15,16]. Taking these factors into consideration, it has been postulated that a more accurate prevalence estimate of CLTI in the United States is between 1 and 3 million [5]. While smoking is a risk factor for large vessel PAD, diabetes mellitus (DM) leads to the progression of small vessel disease. With the rise in DM and an aging population, small vessel CLTI has become increasingly prevalent [17,18]. The extent to which DM leads to the development of CLTI is limited; however, epidemiological studies suggest that the multivariate-adjusted odds ratios are as high as 7.45 [15]. It has been suggested that the probability of amputation is more than 50% higher in DM CLTI patients compared to those without DM [11].

## 2. Current Treatment Procedures

### 2.1. Non-Invasive Assessment

The primary goal in CLTI is timely limb revascularization. Vascular surgeons follow the Rutherford Classification of Chronic Limb Ischemia and the Society for Vascular Surgery (SVS) Wound, Ischemia, and Foot Infection (WIFI) classification system to provide accurate staging [4]. The Ankle Brachial Index (ABI) and Toe Brachial Index (TBI) are non-invasive tests that measure hemodynamics and are used in both classification systems to help stratify the severity of ischemia. After staging, the Patient’s risk, Limb severity, and Anatomic complexity (PLAN) and Global Anatomic Stage System (GLASS) aid in developing an optimal revascularization plan [4]. Often, non-invasive imaging such as Duplex Ultrasound (DUS) and Computed Tomography Angiography (CTA) play essential roles in both PLAN and GLASS as they help define patient-specific anatomy, disease severity, and viable reconstruction options.

### 2.2. Angiography (Diagnostic for Planning a Bypass or Treatment)

In the early stages of CLTI, either diagnostic or therapeutic angiography is employed after an initial workup and DUS. Diagnostic angiography provides the benefit of real-time imaging with the ability to readily intervene and resolve the occlusion. Despite several limitations, angiography remains the gold standard for surgical planning and is an indispensable tool. While angiography continues to provide high-quality vascular imaging, its ability to assess lumen patency, total occlusions, vessel wall characteristics, and vessel sizing is often limited by the catheter’s ability to reach the lesion in question. Furthermore, angiography is limited by its two-dimensional imaging perspective [19]. This can make it difficult at times to determine and plan what intervention is best.

While angiography is less invasive than a surgical procedure, it requires vascular access by a percutaneous puncture in either an antegrade or retrograde fashion. Often, four or five French (0.05 or 0.07 in) catheters over 0.035 or 0.038 in guidewires are used to gain initial access; however, these can be increased in size as needed to accommodate different guide or diagnostic catheters [19]. While rare, complications at the puncture site can result in severe bleeding, requiring blood transfusions or surgical intervention. Angiogram success is strongly dependent on the catheter length; it must be sufficient to traverse the arterial vasculature of the lesion in question. Guide catheter lengths often range from 65 to 100 cm [19]. Selection of a standard mid-length catheter (e.g., 80 cm) may result in a situation where the catheter is too short, requiring multiple catheter exchanges or an inability to reach the lesion in question. Small or micro-guide catheters 5 F (0.07 in) are required to navigate the smaller popliteal and tibial vessels [20]. Unfortunately, these small guide catheters often suffer from an increased lack of support, torquability, and steerability compared to larger guide catheters [19].

### 2.3. Limitation of Current Endovascular Techniques

Endovascular success in re-establishing patency is largely dependent on anatomic complexity, length of the lesion, and extent of tissue loss. While long-term patency is important, the primary goal in treating CLTI is limb salvage and maintaining the patient’s quality of life [21]. The best approach to revascularization, surgical or endovascular, has been debated for some time. The BEST-CLI Trial in 2022 noted that surgical bypass plays an important role in revascularization for patients with an adequate great saphenous vein (GSV) as a potential conduit with a decreased risk of major adverse limb event (MALE) or death in the surgical patient cohort compared to the endovascular group (42.6% vs. 57.4%, *p <* 0.001) [22]. However, for those who require an alternative conduit due to an inadequate or absent GSV, the risks of developing MALE or death are similar for endovascular and surgical bypass approaches (43.8% in the surgical group versus 47.7% in the endovascular group *p* = 0.12) [22]. These results highlight the importance of an individualized approach when choosing between surgical and endovascular intervention for revascularization, which requires evaluation of both the arteries and veins, along with other patient factors, including medical comorbidities.

Limitations in endovascular treatment often stem from a combination of patient and technical factors. The complexity of wire and catheter navigation plays a large role in limiting endovascular treatment. Small, tortuous, stenotic, and fragile infra-popliteal vessels are difficult to traverse. Current 5–7.5 F (0.07–0.1 in) microcatheters and wires lack the precision that larger 8.5 F (0.11 in) [23,24] steerable catheters possess in cardiovascular and aortic angiography. They are often straight, single-lumen, and unable to be manipulated in real time. This lack of precision produces an inability to steer to chronic occlusions of the tibial vessels.

Current methods for traversing infra-popliteal arterial lesions include antegrade guidewire crossings or retrograde tibial and pedal approaches; the latter is often used if antegrade crossing fails or subintimal entrapment occurs [25]. Commonly used catheters like the CXI support catheter (Cook Medical, Bloomington, IN, USA) on 0.035, 0.018, or 0.014 guidewires face challenges in steerability, limiting evaluation of the lesion and treatment of tortuous infrapopliteal disease. Less frequent salvage techniques include re-entry devices for subintimal recanalization, subintimal arterial flossing with antegrade–retrograde intervention (the SAFARI technique) [26], and “double-balloon” approaches for subintimal plane communication. While most reentry devices are too large for infra-popliteal use, lower profile options like the Enteer (Medtronic, Minneapolis, MN, USA), Ocelot (Avinger, Redwood City, CA, USA), and STINGRAY LP (Boston Scientific, Marlborough, MA, USA) are available [25]. Each device presents risks, including arterial dissection and perforation, as do guidewire crossings, which can cause arterial wall perforation, subintimal entrapment, and distal embolization [25,27]. These devices have many challenges. Furthermore, currently available devices do not have the ability to assess the lesion and determine the best treatment plan for the patient. This can lead to additional imaging, procedures, and complications.

## 3. Introduction to Steerable Catheters

While steerable catheters are not commonly used in small peripheral vessel vascular disease, they are frequently employed in other procedures [24] such as complex cardiac [28], renal [29,30,31], and aortic procedures [23,32]. Studies show that introducing true steerability into large catheters reduces failure rates, procedural time, fluoroscopy time, and contrast use compared to conventional catheters [24,25]. It is important to note that “steerability” is also used as a marketing term for catheters and guidewire flexibility in some systems, such as Boston Scientific’s Fathom^TM^ Steerable Guidewires system [33]. In these instances, it is used to describe greater flexibility about a selected axis. Throughout our paper, steerability here refers to the user’s ability to actively reorient the catheter’s tip instead of a passive increase in bendability about some directions that some clinically available devices provide.

Often a steerable catheter is utilized in the selection of arterial branches directly off the aorta, such as the superior mesenteric artery (SMA). The enhanced navigation control of the catheter facilitates the selection of the SMA and reduces radiation exposure to the patient compared to catheters that lack steerability [34,35]. The steerability of the catheter reduces the possibility of injuring the aorta since steering enables precise navigation. However, the catheters involved in these aortic procedures are quite large and bulky [36], making them unsuitable for tibial, cerebral, and other small-diameter vascular beds.

Large steerable catheters are also utilized in patients with abdominal aortic aneurysms (AAAs) via endovascular repair (EVAR). During EVAR, the delivery catheter stores the stent graft and is navigated under X-ray guidance [32]. Once the catheter reaches the desired landing zone, the stent graft is released from the catheter, excluding the aneurysm and functioning as the new vessel wall [32]. Prototypes of a steerable electromagnetic (EM) trackable catheter for endovascular aortic repair have shown to reduce radiation exposure [37]. Produced by DEAM (Amsterdam, The Netherlands), the 90 cm catheter provided access to the renal and superior mesenteric arteries in three pigs. The tip of the catheter is able to bend up to 90 degrees and rotate in a full circle via the use of a distal controller. Fusion imaging technology has been used in the mapping of the aorta and neighboring vessels via the clinically available Cydar and Philips catheter systems, reducing the need for radiation and contrast agents [38]. Using artificial intelligence to process the Cydar catheter’s navigational data, a three-dimensional model can be created, aiding procedural planning and interventional decision making [38]. Besides abdominal aortic aneurysms, steerable catheters are also frequently used in aortic valve replacements [39]. A catheter is needed to steer across the aortic arch in order to place the valve in the desired location. Injury to the aorta can result in massive hemorrhage requiring emergent conversion to open surgery with high morbidity and mortality.

Advancements in neurovascular microcatheters have led to substantial improvements in their navigational precision and steerability. Recent studies indicate that modern microcatheters are designed not only for navigation through small-diameter vessels but also for enhanced directional control. For instance, steerable microcatheters with hydraulic actuations integrate a bioinspired soft robotic device at the catheter tip [40]. The utilization of hyperelastic soft materials facilitates its precise tip bending and steering [40]. This steerable microcatheter for neurovascular intervention is promising as it reports a faster targeted vessel selection and decreased procedure time [41,42].

Unfortunately, the utilization of microcatheters for the endovascular treatment of small vessel peripheral arterial disease (PAD) has been essentially the same since the mid-1990s despite advancements in other endovascular fields [24]. While other re-vascularization options exist, such as intravascular lithotripsy via the FDA-approved Shockwave E8 Peripheral IVL Catheter system [43], or transcatheter venous arterialization via the Lim Flow device [44], these systems are unable to provide diagnostic information and assessment to help guide treatment planning for the patient. Steerable microcatheters would not only provide diagnostic information, but also improve patient and procedural outcomes [45].

## 4. Mechanics of Steerable Catheters

The concept of a steerable catheter encompasses magnetic catheters, smart material catheters, pull–push systems, electromechanical actuation, and hydraulically actuated devices [46,47], each conceptually distinguished by the different engineering designs employed to achieve precise steering control. Steerable catheters encompass magnetically actuated devices [48,49], smart material catheters [50], push–pull Bowden systems [24], electromechanical actuations [51], and hydraulic actuation [40]. There are two components to the catheter system: the operator interface (i.e., for the surgeon or *operator*) and the patient interface (i.e., deflectable tip within the patient) [52]. If designed properly, each interface aids the operator in making safe and useful changes in the catheter’s 1–2 cm tip shape. Furthermore, the design of the operator interface as a handheld device allows for intuitive changes with—hopefully—forces that reduce the risk of occupational hand injuries [53]. The patient interface encompasses the catheter and ancillary equipment (the sheath, guidewire, and other tools) that collectively interact with the patient’s body. Under radiation guidance or fluoroscopy, the interventionist can visualize the location of the catheter within a blood vessel, typically using fiduciary markers. For example, gadolinium beads [40] can help with steering and navigation to the treatment site. Once ideally positioned, the steerable catheter provides stability during the delivery of medication, coiling wires, or stents, helping to prevent the dislodging of the tip that requires painstaking repositioning before continuing the procedure.

Researchers and catheter manufacturers have considered or adopted a variety of means to improve the safety and precision of catheter tip deflection, with gradients in the stiffness of the catheter along its length [54,55]. An example of this is the alteration of the PEBAX material properties, from proximally stiff to distally soft that is used in the Fathom^TM^ Steerable Guidewires system [33]. For example, in neurointervention, it is typical to nest small-gauge flexible catheters, within larger-gauge, stiffer catheters to improve navigation through larger vessels to those within the. skull [40,56]. Researchers have furthermore considered hydrophilic lubricating coatings [57] to reduce the friction present between the arterial wall and the advancing catheter, in turn helping to reduce the risk of elastic energy accumulation and “kickback” (or sudden release), which can lead to devastating vascular injury. Compared to conventional catheters, steerable catheters have produced a useful reduction in procedure time [41,58,59].

## 5. Push-Pull Method for Steering Catheters

**Figure 2:**
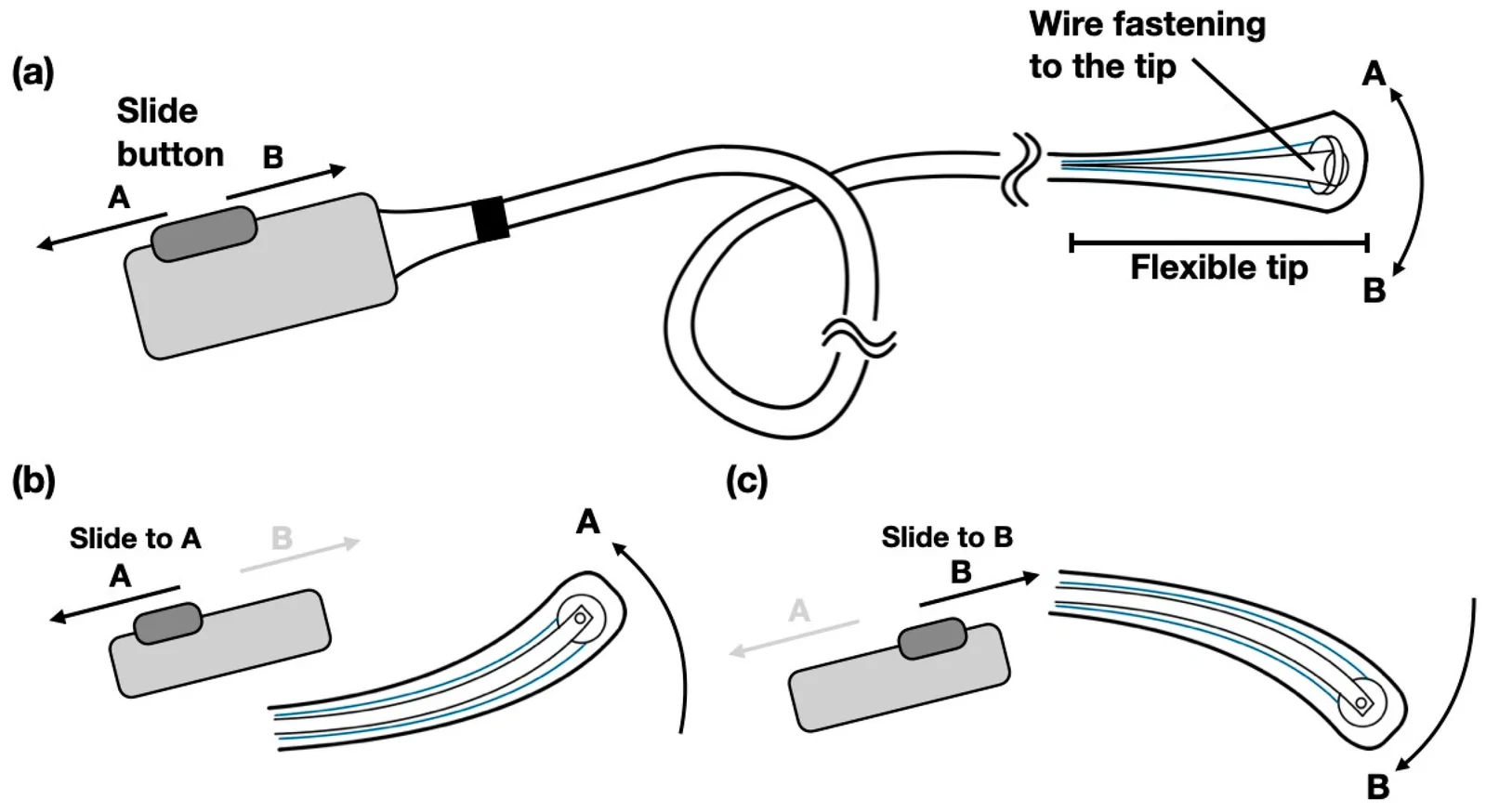
(**a**) Schematic illustration of the push–pull catheter system. The catheter tip remains straight when the button is in a neutral position. (**b**) When the button is moved to position A, it enables the flexible tip to bend toward direction A. (**c**) When the button is moved to position B, it enables the flexible tip to bend toward direction B.

Steerable push–pull catheters adopt the Bowden cable approach [24] that was originally used for bicycles and later in automotive applications. By providing axial displacement of one or more wires encased in a housing that prevents radial deflection or buckling of the wires and axial stretch of the catheter (Figure 2), one can transmit motions over long distances from the proximal operator’s control to the distal tip. Through differential axial motion, bending of the tip can be accomplished. The requisite stiffness of the housing makes it difficult to design catheters with this approach for applications in fragile, small, or tortuous vasculature. Moreover, ample lubrication is required within to ensure facile steerability [42]. Finally, bending the catheter causes differential axial displacements in the enclosed wires, causing the tip to be deflected without commanded changes from the operator, complicating its practical use. This is especially prevalent when the catheter also encloses a lumen to pass tools to the treatment site. These issues contribute to a greater risk of intima damage during use of this method. The Agile Devices Angler^TM^ is an example of a push–pull system with a maximum outer diameter of 3 F (0.04 in) used in peripheral and coronary vascular procedures [60]. The 1.5 cm long steerable tip is covered with a silicone coated coil to enable deflection up to 180 degrees via control from the operator interface [60]. Some push–pull system-based microcatheters have a bioinspired bevel-tip needle (PBN)—composed of four probes—that is scaled down for micro-level application using thermal drawing, a process where heated polymer is drawn into thin, sub-millimeter fibers [61]. Another example of a push-pull catheter utilizes laser-cut nitinol tubes, patterned to correspond with the steerable segment of the outer sheath [62]. The bending of the catheter tip is achieved through the push-and-pull mechanism of the nitinol wire [62]. While this approach is effective for endoscopic submucosal dissection, scaling the laser-cut design for peripheral vascular interventions presents significant challenges due to electrostatic 252 interactions and other interfering and significant forces during fabrication and assembly [63].

## 6. Hydraulic Method for Steering Catheters

**Figure 3:**
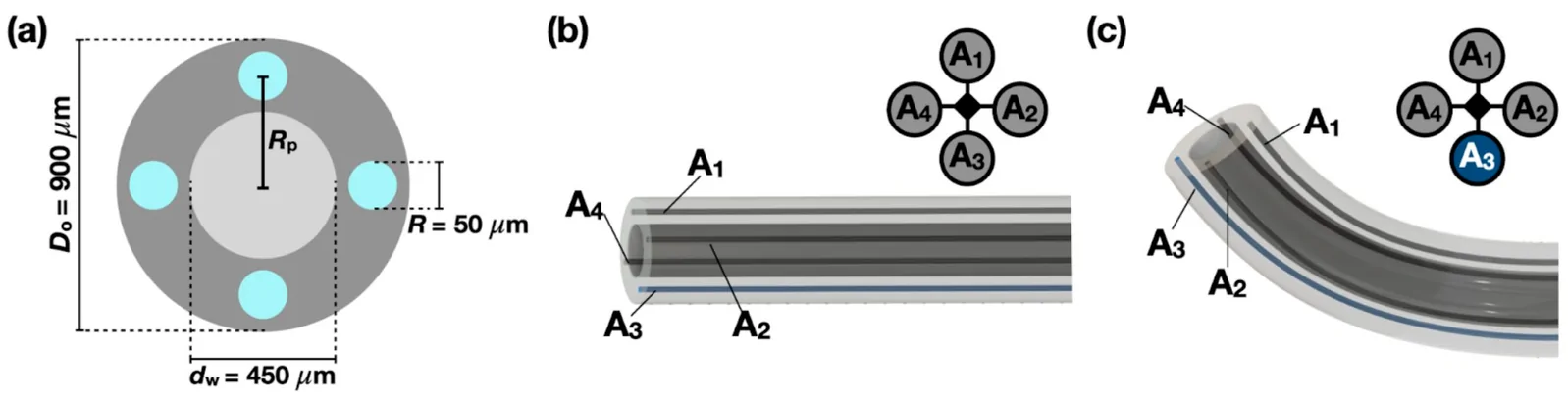
(**a**) A display of a cross-sectional view of the catheter tips. The central lumen is reserved for the guidewire, while the four surrounding channels (cyan) are used for deflection through the introduction of pressure via saline as a working fluid. (**b**) An illustration of the native state of the catheter tip when no saline is injected into the other four saline channels (A_1_, A_2_, A_3_, A_4_). (**c**) During actuation, the catheter tip deflects upward when saline is injected into the A_3_ saline channel (blue).

Hydraulic actuation was commonly perceived to be difficult due to the combination of long working distances of 1.5 m or more and the small fluidic passages at a diameter of 100 µm or less. The discipline of *soft robotics* [64] was largely founded upon the use of air as a working fluid to compensate for this perceived issue. However, gas is not an option in endovascular intervention due to the risk of device failure leading to an embolism [65]. For this reason, hydraulic actuations with sterile saline as a working fluid is the only reasonable alternative. At first glance it would seem that hydraulic catheter devices would be impossible, as the viscous forces would overwhelm the ability to pass fluid along such passages with mere hand force due to the principles of microfluidics. However, as an incompressible fluid, pressure is easily transmitted in these conditions because little axial flow is required to produce significant pressure changes along the passages. This is made clear from the routine use of micro balloons in endovascular procedures for the introduction of stents and placement of coiling wires [66]. Coupled with the ability to amplify the forces produced by the operator’s fingers via a relatively large fluid cavity in the operator’s interface (i.e., using Pascal’s principle), significant deflections are enabled by small forces from the operator’s fingers. An issue that arises from the use of hydraulics in flexible steerable tips is the tendency of the tip to radially expand when pressure is introduced. Axial expansion of, for example, A_3_ (as seen in Figure 3), produces a tip deflection, but the fluid passage can also radially expand, swelling the catheter and potentially causing a dangerous occlusion of the blood flow in the confined space of the parent vessel or within a lesion. Coating the catheter with a thin but relatively rigid polymer layer has been shown to overcome this issue, as has the use of a stress-stiffening polymer [40]. The radial expansion occurs over a much shorter length scale, and consequently, the strain increases far more rapidly in this direction. By using stress-stiffening materials, the radial displacement can be reduced for a desired axial displacement that itself is responsible for the tip’s deflection. An additional benefit to the hydraulic approach is the ability to introduce variable stiffness: one can raise the pressure in all fluid channels to stiffen the structure and hold it in place. This is especially useful for introducing coiling wires into cerebral aneurysms, for example [40]. Navigating to the lesion requires passing through the fragile neurovasculature, where a soft tip is preferred. Coil deployment, by contrast, requires a stable guide for the introduction of the coils into the aneurysm from the main catheter present in the parent artery.

## 7. Magnetic Field Method for Steering Catheters

**Figure 4:**
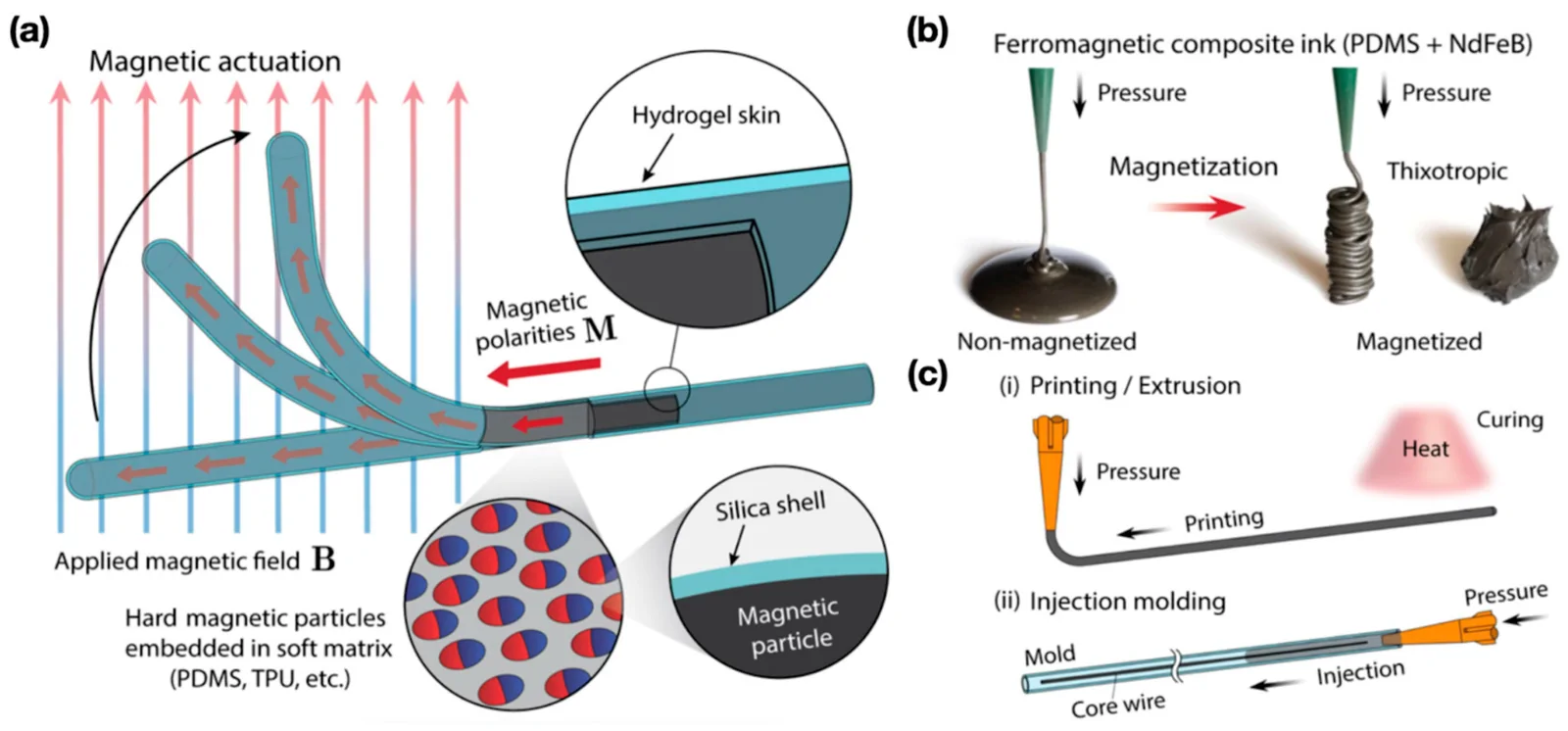
(**a**) An illustration of the catheter tip deflection in the same direction as the direction of the external magnetic field [48]. (**b**) The non-magnetized ferromagnetic composite ink composed of a mixture of NdFeB and PDMS is magnetized [48]. (**c**) An illustration of the extrusion method and injection molding can produce a magnetized catheter tip with a uniform diameter [48]. Reproduced/modified from Figure 1A–F of Kim et al., Science Robotics, 10.1126/scirobotics.aax7329 (2018), AAAS [48].

As human tissues are permeable to magnetic fields, it is possible to use magnetic fields for the actuation of catheter devices, as first proposed in the early 1950s [67]. Magnetic-based steerable catheters have conventionally been used in larger vessels [42], though recent advancements have produced examples with a smaller outer diameter of typically less than 3.0 mm [68]. The steering requires careful magnetic field generation, because the force generated by a magnetic field is at a right angle to the field itself, and because the coils contain inductance that resists changes in the commanded magnetic field [42]. Moreover, a simple analysis shows that the angle of catheter tip deflection is proportional to the size of the catheter tip. Kim et al. [48] proposed a magnetic-based catheter with a solidified mixture composed of magnetized NdFeB particles with uncured PDMS resin or TPU in an optimized proportion shown in Figure 4. The catheter tip was prepared by injection molding or extrusion of a mixture, forming a catheter tip with a uniform diameter. To steer the catheter, an external magnetic field was applied upon the catheter tip and deflects the tips about an axis parallel to the applied magnetic field.

## 8. Piezoelectric and Smart Material Methods for Steering Catheters

### 8.1. Micromotors and the Like for Catheter Steering

The application of piezoelectricity and ultrasound has been proposed and primarily explored for cerebrovascular conditions [69,70,71], demonstrating potential for peripheral vascular interventions due to the similar size as previously suggested. Such an application enables the catheter to produce translation and rotational motion. Yun et al. [69] introduced an ultrasonic micromotor capable of multi-degree-of-freedom motion. When a sinusoidal voltage potential is applied at a systematic resonance frequency, it deforms the lead zirconate titanate (PZT) element, causing transverse bending across the catheter’s cross-sectional surface. Applying an electrical signal at the resonance frequency causes vibration sufficient to drive motion of the tip, and controlled vibration along two perpendicular axes enables precise catheter steering in any desired direction [69]. Compared to other microcatheter designs, such as hydraulic or magnetic actuators, micromotor-based actuators offer a shorter response time [69]. However, they suffer from complexity and the presence of components that, if lost, could injure a patient.

### 8.2. Optically and Electrically Expanding Polymer Media for Catheter Steering

Smart material actuators offer a promising alternative for microcatheter steering. For example, nitinol, a shape-memory alloy composed of nickel and titanium, retains a predefined shape after cooling from its activation temperature once formed into the desired configuration [72]. The Columbus steerable guidewire (Rapid Medical, Yokneam, Israel), constructed from nitinol, is designed to navigate brain vessels for the treatment of aneurysms [73]. However, the application of smart materials in catheter tips is often constrained by environmental conditions, such as temperature [74]. Lee et al. [75] proposed a fabrication method for a dielectric elastomer actuator to enable microcatheter steerability when voltage is applied. This catheter tip integrates two multilayers of dielectric elastomer actuators, with polydimethylsiloxane (PDMS) selected as the elastomer layer due to its biocompatibility [76]. By applying varying voltages, the catheter tip bends at different angles, allowing for the selection of specific arteries—a functionality similar to magnetic field and hydraulic actuation approaches.

## 9. Challenges in Producing Steerable Catheters

Developing a steerable catheter for treating small peripheral vascular disease is challenging due to the precise scale required for the catheter tip’s geometry. Fabrication methods have advanced for years, allowing the machining of components for arbitrary shapes down to the microscale [77] and even the nanoscale [78,79]. Many commercial steerable catheters rely on conventional mechanical systems, where thin wires actuate the steering through antagonistic mechanisms [80,81]. This approach presents significant challenges when attempting to miniaturize the mechanism to a microscale [82,83], thereby limiting its applicability for tibial vessel treatment.

Magnetic continuum robots with steering capabilities offer an alternative to traditional wire-pulling mechanisms [84,85]. Recent advancements in magnetic steerable catheters, where magnets are encapsulated within soft materials, have demonstrated potential for application in cardiac interventions, particularly within coronary arteries [86]. However, integrating magnets presents risks, including the possibility of the magnets dislodging from the soft material, which could lead to complications during procedures [48]. Wu et al. [49] are attempting to create a steerable catheter for PAD treatment that is magnetic-actuation-based with location sensing; however, the maximum angle of deflection is 40°. The accessibility of this steerable catheter is limited compared to steerable catheters with soft robotic actuation, with a maximum angle of deflection of 90° [40]. Other challenges in catheter design include a balance between rigidity and flexibility such that the catheter provides both steering and stability [84]. 

Advancement in photolithography enables the time and cost-efficient fabrication of microfluidic devices [87,88,89] and semiconductor circuits [90,91,92]. However, a steerable catheter requires a soft and deformable material at the tip to enhance dexterity and minimize the risk of vascular injury. Moreover, manufacturing and machining using hyperelastic materials such as silicone on a scale ranging from 10 µm to 1000 µm is extremely challenging due to interactions from van der Waals, electrostatic, and gravitational forces [63]. Therefore, researchers are shifting their micro-fabrication methods to techniques like flow casting, flow-governed layer casting, and peel-dominated demolding, which are more effective for creating intricate microstructures such as detailed blood vessels [93,94]. Given the intricate details and scale required for the catheter tip, any error can lead to fabrication failures, such as nonuniform thickness distribution, oversized molding, or the inability of the 3D printer to capture fine details [95]. Table 1 summarizes the different types of steerable catheters, highlighting their unique advantages and limitations as well as their corresponding geometric characteristics.

## 10. The Benefit of Steerable Catheters in CLTI

Endovascular therapy in CLTI is emerging as the first line for disease management, either diagnostic or therapeutic. The advent of steerable catheterization enhances outcomes in those who otherwise may not be eligible for a surgical bypass or other surgical revascularization procedures. Large steerable catheters have shown promise in large vessel occlusions by potentially reducing procedural time [41], lowering radiation exposure [41,102,103], extending access to previously unreachable target sites [52,104,105], and providing other significant benefits [52] such as precise therapeutic drug delivery. Below, we will highlight the benefits of a steerable catheter for CLTI.

### 10.1. Enhanced Navigation and Access

**Figure 5:**
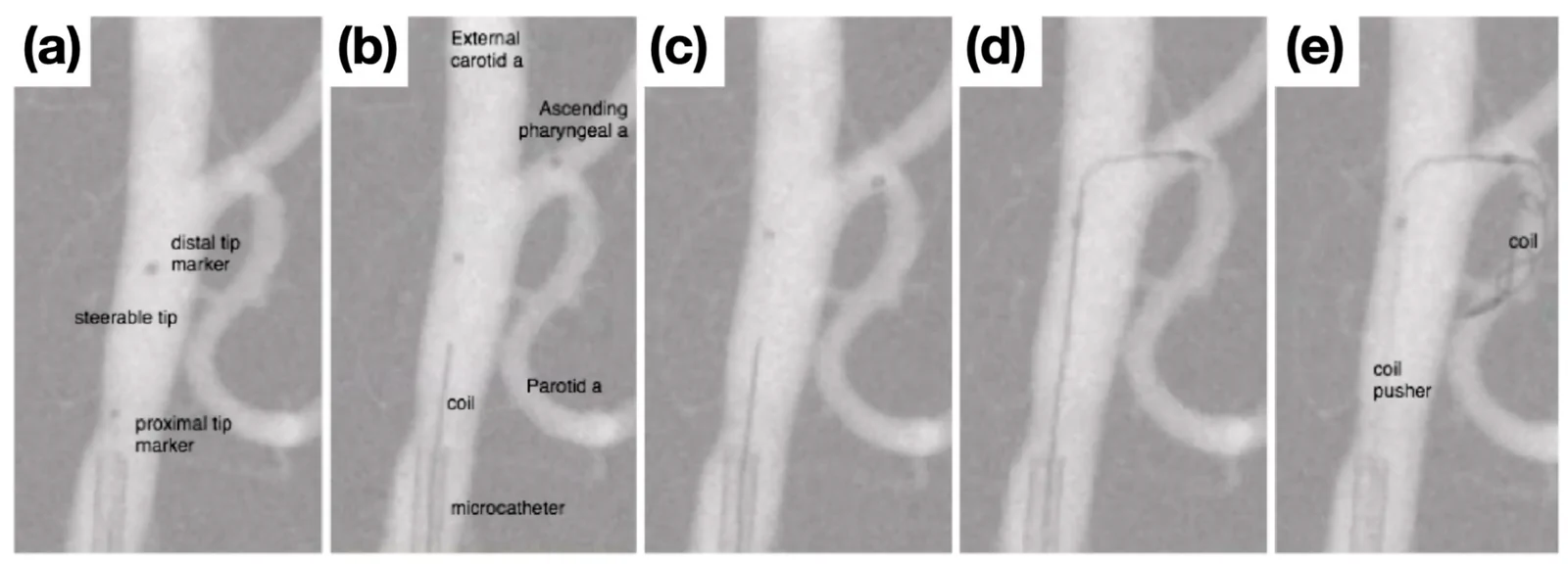
(**a**–**e**). An illustration of how a steerable microcatheter tip produces a 180° turn from the parent artery [40]. Reproduced/modified from Figure 6f–j of Gopesh et al., Science Robotic, doi: 10.1126/scirobotics.abf0601 (2021), AAAS [40].

Steerable catheters, which enhance the surgeon’s dexterity in another vascular procedures, could offer similar benefits for catheterization in tibial vessel CLTI patients. The recent literature suggests that catheters with a constant angle of deflection allow surgeons to rotate them to navigate tortuous arteries [106]. Although a bent tip allows the interventionist to rotate the catheter in the desired direction, it occupies extra space, making navigation through narrow vessels challenging. Additionally, rotating the catheter adds time to the procedure. A steerable catheter addresses these issues by allowing interventionists to deflect the tip only when necessary, providing precise control without occupying extra space. This implies that steerable catheters could provide this valuable advantage, especially for crossing lesions in the tibial segment [107]. Figure 5 demonstrates the use of a steerable microcatheter in neurovascular procedures, illustrating the potential applicability of this technology to tibial artery-related conditions due to similar arterial geometry and characteristics [40].

Conventional catheters have higher amounts of wall injury (arterial intima injury) than steerable catheters due to increased catheter tip and vessel-wall interactions. The catheter tip often interacts with the vessel wall when the vessel begins to turn at an angle. The lack of steerability inhibits the turning and increases the risk of penetrating the vessel wall [108]. Comparatively, the steerable catheter tip provides significantly greater operator freedom than a conventional catheter, reducing the number of interactions between the catheter tips and the vessel wall, in turn reducing the risk of vessel injury [109].

### 10.2. Precision in Therapeutic Delivery

Therapeutic delivery encompasses the administration of thrombolytics, vasodilators, anticoagulants, and other pharmacological agents directly to the pathological site [110]. Additionally, recent advancements have introduced biomaterial-based carriers designed to protect gene therapies—comprising reactive nucleic acids—from degradation, thereby enhancing the modulation of gene function within the targeted region [111]. Catheter intervention enables therapeutic delivery, reduces patient discomfort, and minimizes physiological change [112]. However, many studies neglect the accuracy of catheter tip placement as an evaluation parameter in gene delivery, which can significantly impede the effectiveness of drug delivery [113].

Another example of therapeutic drug delivery in PAD is catheter-directed thrombolysis. In the setting of arterial occlusion, if the catheter tip is unable to reach the origin of the occlusion, precise delivery of thrombolytics is challenging. The more selective drug delivery approach has been shown to produce higher efficacy and decrease bleeding complications [114]. In a systematic review of catheter-directed thrombolysis protocols by Ebben and colleagues, lower thrombolytic treatment doses were accompanied by decreased incidence of major and minor bleeding complications [114]. While it was noted that lower doses of thrombolytics were associated with longer treatment duration, it is unclear how selective these treatments were in regard to the proximity of the catheter tip to the origin of the occlusions [114]. It is reasonable to predict that closer proximity of the catheter tip to the origin of thrombosis may have a positive impact on plaque lysis and that the treatment dosage and duration required may be reduced, improving patient outcomes.

### 10.3. Potential to Improve Patient Outcomes

The importance of catheter tip placement, steerability, and design advancement has been noted to be a topic of significance in specialties such as interventional cardiology and neurosurgery. This is due to the many potential benefits and improved patient outcomes that have been reported [24,40]. Increased steerability can reduce operator error, catheter deflection, and procedural complications while enabling increased navigation through challenging and tortuous vasculature [61]. Decreasing torque can allow for better maneuvering of the catheter tip. This increased control can decrease the rate of unintended tip injuries that can lead to subintimal and branch vessel damage, dissection, perforation, post-intervention restenosis (PRIS), and in-stent restenosis (ISR) [24]. Improved catheter tip control can also lead to increased precision in drug delivery, resulting in maximizing drug therapy and reducing complications such as toxicity and bleeding. In addition to patient safety, steerable catheters have been shown to reduce procedural time amongst interventionists in cardiology and electrophysiology as well as permitting treatment in cardiac patients that otherwise would be unsuccessful using basic trans-catheter approaches due to inferior navigation, positioning, and stability of these catheters [45,115]. These catheter benefits are not exclusive to these specialties alone, and by creating a specific micro-steerable catheter for distal tibial arterial disease, vascular surgery is likely to also see similar improved patient outcomes.

### 10.4. Limitations

Limitations to our review include that it is a narrative review and not a systematic review with a formal search strategy. This review is not all-encompassing and thereby may have inadvertently omitted relevant literature. It has a predominance of preclinical/animal and adjacent-specialty evidence due to the lack of current prospective clinical trials in this topic area. This may make conclusions weaker or less effective than if performed within the same specialty, and at times may result in the inability to draw comparative-effectiveness conclusions.

## 11. Summary

Our review highlights the need for steerable microcatheters in tibial vessel intervention. Current research shows that CLTI is increasing, and current practices in CLTI are not eliminating patient morbidity or mortality, let alone the significant economic burden on the medical health system. Steerable catheters in larger vessels have been shown to improve treatment with decreased procedural complications, procedural times, and increased precision in drug delivery. Despite these endovascular treatment advancements in large vessels, the ability to create steerable microcatheters that can traverse tortuous and stenotic vessels in tibial vessels has been somewhat limited. Many of the challenges are a result of needing to miniaturize the devices to the microscale. A critical component of these steerable catheters is the geometry of their tip and the ability to have flexible deflection. Advancements in micro-fabrication methods have shown early promise in creating intricate microstructures; however, challenges in precise printing remain. Further research and development of novel microcatheters is needed. Refinement of steerable microcatheters in tibial vessel intervention would tremendously expand current endovascular capabilities both in treatment and diagnostic planning. Further prospective trials would be beneficial in further delineating specific outcomes. Regardless, it is reasonable to assume that microcatheters in tibial vessel intervention would likely translate to an increased positive impact on patient care, especially in a historically disadvantaged patient population.

## Figures and Tables

**Table 1:** A comparison of methods used to actively steer endovascular catheters.

Design	Outer Diameter	Pros	Cons
Push–pull system	0.8–4 mm [96,97]	Completely mechanical design	-
Hydraulic actuation	0.25–0.9 mm [40]	Flexible, reduced chance of vessel injury, variable stiffness	Stiff structure [42]; Complex fabrication, radial expansion, nonlinear deformation causes control challenges [98]
Magnetic-field actuation	2.7 mm [75]	High precision achieved by multi-degree-of-motion [99]	Challenging field control, complex design [100]; Risk of losing the actuator when interacting with the vessel wall [42]
Piezoelectric and smart material methods	1.4–4.8 mm [68]	Accurate steering with developed algorithm [48,101]	-

## Data Availability

No new data were created or analyzed in this study. Data sharing is not applicable to this article.
